# Graphene-Enhanced Raman Scattering from the Adenine Molecules

**DOI:** 10.1186/s11671-016-1418-5

**Published:** 2016-04-14

**Authors:** Leonid Dolgov, Denys Pidhirnyi, Galyna Dovbeshko, Tetiana Lebedieva, Valter Kiisk, Siim Heinsalu, Sven Lange, Raivo Jaaniso, Ilmo Sildos

**Affiliations:** Institute of Physics, University of Tartu, W. Ostwald st 1, Tartu, 50411 Estonia; Institute of Physics, NAS of Ukraine, Prospect Nauky 46, Kyiv, 03028 Ukraine

**Keywords:** Graphene, Raman scattering, Chemical enhancement, Adenine, 78.30.-j, 78.67.-n, 78.67.Wj

## Abstract

An enhanced Raman scattering from a thin layer of adenine molecules deposited on graphene substrate was detected. The value of enhancement depends on the photon energy of the exciting light. The benzene ring in the structure of adenine molecule suggests π-stacking of adenine molecule on top of graphene. So, it is proposed that the enhancement in the adenine Raman signal is explained by the resonance electron transfer from the Fermi level of graphene to the lowest unoccupied molecular orbital (LUMO) level of adenine.

## Background

Non-invasive sensing of biological molecules, especially deoxyribonucleic acid (DNA) and its constituents, by means of label-free optical spectroscopy can open new prospects in biomedical analysis. Recently, we described a possibility for fluorescent detection of low concentrated DNA solution infiltrated into globular photonic crystal [1]. Since the fluorescence of DNA and its constituents is comparatively weak, Raman technique instead of fluorescence was used for detecting low concentration of adenine in solution. Adenine is one chemical component of the DNA.

Maximal theoretical enhancement of the Raman signal caused by the surface of nanostructured noble metals can reach tens of orders of magnitude [2]. Such significant enhancement is usually associated with local concentration of electric field in the vicinity of metallic nanostructures because of surface plasmons and referred to an electromagnetic mechanism of surface-enhanced Raman scattering (SERS) [3]. However, realizing such an enhancement in practice is not very usual. Thus, in the case of adenine, moderate Raman enhancements have been reported by different scientific groups both for the adenine adsorbed onto silver electrodes [4, 5] and for the solid adenine films deposited onto nanostructured aluminum surface [6]. In the latter work, 1-nm-thick layers of adenine molecules were deposited on nanostructured aluminum surface and the reference fused silica substrate. The fact that the authors of [6] could detect the presence of such thin adenine film on the fused silica substrate testifies that the sensitivity of their setup was on the high side. But, the enhancement of the Raman signal caused by nanostructured aluminum was only seven times. Recently, Mevold et al. [7] probed graphene covered by gold nanoparticles as SERS substrate for adenine and obtained a threefold increase in Raman intensity. In that work, graphene was used only as a substrate for assembling gold nanoparticles. Small benefit in SERS was associated not with graphene but with plasmonic effect caused by the gold nanoparticles.

Application of graphene as a substrate for SERS has renovated the interest in the use of surface-enhanced spectroscopy in optical sensing. It has been established that the Raman signal from some organic molecules deposited on graphene can be enhanced but usually less than in the abovementioned plasmonic case. The effect itself is quite novel for graphene and depends both on the type, spatial orientation of the tested molecules, strength of their interaction with graphene, molecular energy levels, and graphene properties [8, 9]. Graphene-enhanced Raman scattering is associated with a charge transfer from graphene to the tested molecules, which is also called the chemical mechanism of SERS. Current research is directed towards determination of molecular selectivity and sensitivity limits at which Raman scattering from the tested molecules can be enhanced by graphene [10]. The main benefit of using graphene instead of noble metal is its ability to spectrally separate manifestations of Raman chemical enhancement in the visible light region from electromagnetic enhancement inherit to the far infrared range [11]. For the noble metals, the effects of chemical and electromagnetic enhancements are spectrally overlapped.

In the present work, we report about up to 12 times enhancement of the Raman signal from an adenine layer on graphene in comparison with adenine deposited on quartz substrate and discuss the reasons for such effect.

## Methods

**Fig. 1:**
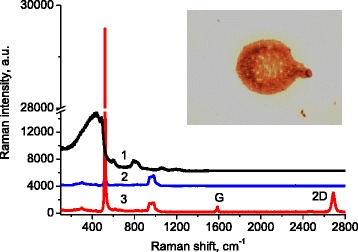
The Raman spectra of silica (*1*), silicon (*2*), and graphene on silicon (*3*). Spectra are not normalized but only shifted along the vertical axis for clarity. *Inset*: microscopic image of a dried micro-droplet of adenine solution deposited on the graphene surface

We used wide-area commercial graphene sheets prepared by chemical vapor deposition and transferred on a silicon substrate covered with a 200-nm-thick silica layer. The adenine was dissolved in distilled water at a low concentration (0.1 mg per ml). This solution was deposited on graphene samples as microliter droplets. Hydrophobicity of the graphene surface and the low concentration of adenine provided homogeneous drying of the small droplets in the form of thin films (Fig. 1, inset) avoiding ruptures and rings associated with regular drying of solutions [12]. Similar droplets were deposited also on the top of specially hydrophobizated quartz substrates. Intensities of the Raman signals from the droplets dried on the graphene and quartz substrates were compared.

Raman spectra from the droplets were measured on a Renishaw inVia micro-Raman setup equipped with a multiline argon laser using either the 488- or 514-nm laser line for excitation.

## Results and Discussion

In order to distinguish Raman bands of adenine from those inherent to substrate, we measured and plotted the Raman spectra of bare silica (Fig. 1, spectrum 1), silicon (Fig. 1, spectrum 2), and graphene on silicon substrate (Fig. 1, spectrum 3).

The Raman spectrum of silica substrate (Fig. 1, spectrum 1) is determined by known SiO_2_ vibrations, which are described, for example, in references [13–16].

The Raman bands of silicon at 520 and 950–1000 cm^−1^ (Fig. 1, spectrum 2) are associated with the first- and second-order Raman scattering from the optical phonons of Si lattice [17, 18]. The presence of the G band at 1580 cm^−1^ and 2D band at 2700 cm^−1^ (Fig. 1, spectrum 3) indicates graphene layer on the top of silicon. The ratio of the two bands implies the presence of single-layer graphene. The nature of the graphene band at 2450 cm^−1^ is debatable, for example, Ferrari and Basko [19] assign this band with a combination of a D phonon and a phonon belonging to the LA branch.

**Fig. 2:**
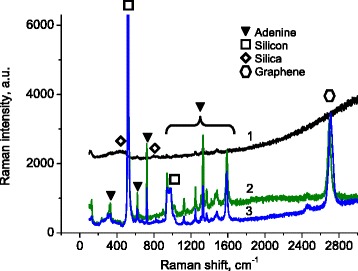
Raman spectra of dried adenine droplets deposited on quartz (*1*) and graphene (*2*, *3*). Excitation wavelengths used are 514 nm (for spectra *1* and *2*) and 488 nm (for spectrum *3*). Spectra are not normalized but only shifted along the vertical axis for clarity

The measured Raman spectra of adenine droplets dried on the graphene-coated substrate (Fig. 2, spectra 2 and 3) contain both bands typical for solid adenine and 2D band related to the underlying graphene as well as bands associated with silicon at 520 and 950–1000 cm^−1^ and group of silica bands (Fig. 2, spectrum 1). The slope of spectra 1 and 2 in Fig. 2 is a manifestation of adenine background fluorescence. Since graphene is known [8, 9] as a quencher of fluorescence, the slope of spectra for the adenine on graphene is less pronounced. It is hard to distinguish the position of the G band of the graphene coated by adenine because it is overlapped with one of the adenine bands. The spectral positions of the adenine Raman bands are in good agreement with literature data (see reference [20]).

One can see that the Raman scattering from the adenine deposited on graphene is up to 12 times stronger than that from the adenine deposited on quartz substrate.

**Fig. 3:**
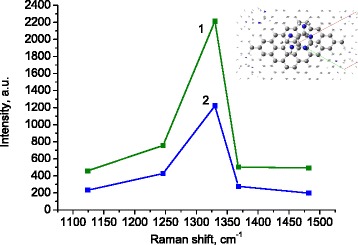
The intensities of the adenine Raman bands in the range 1100–1500 cm^−1^ at the cases of 514-nm (*1*) and 488-nm (*2*) excitations. Intensities are background corrected and normalized to incident laser power. *Inset*: schematic arrangement of adenine molecule on the graphene surface. Nitrogen atoms are marked by a *blue color*

The structure of the adenine molecule provides a possibility that the benzene ring of adenine can be overlapped with the carbon ring of graphene (Fig. 3, inset). Such structure named as π-stacking enables contact of the π-electron shells of graphene and overlying adenine molecule. We suspect that in exactly that relative position, one can expect favorable conditions for resonant electron transfer from the Fermi level of graphene to the lowest unoccupied molecular orbital (LUMO) level of the adenine molecule leading to an enhancement of the adenine Raman signal.

This is a specific example of the so-called chemical mechanism of Raman enhancement [8–10]. It works if the photon energy of the exciting light is resonant with the energy gap between the Fermi-LUMO levels. The first judgment about this resonant mechanism can be done if the intensities of Raman scattering will be compared at different excitation wavelengths. Comparison of spectra 2 and 3 measured with 514- and 488-nm excitations (Fig. 2) shows that the corresponding Raman signal excited at 514 nm is slightly stronger. This is illustrated in Fig. 3, where the intensities of the most pronounced adenine Raman bands in the range 1100–1500 cm^−1^ are plotted after subtraction of the base line and normalization on the laser intensity for both excitation wavelengths.

It can be speculated that the light with photon energy 2.41 eV (514 nm) fits better to the energy gap than the photons with energy of 2.54 eV (488 nm). The complication here is in the identification of the exact LUMO and highest occupied molecular orbital (HOMO) energies of the adenine molecule with respect to the vacuum level taken as a reference point. Experimentally, it can be done, for example, by derivation of the real and imaginary values of the permittivity of solid adenine films in the deep ultraviolet spectral range. It is done, for example, in article [21] by means of using synchrotron irradiation. The value of the HOMO-LUMO gap obtained from these measurements is ~4.4 eV. It roughly corresponds to the value of ~4 eV calculated by the use of the density functional theory [22, 23]. Similar calculations have been made in reference [24]. There, the HOMO-LUMO gap is reportedly 3.8 eV, and the relative position of the LUMO and HOMO levels of adenine is mentioned as −2.2 and −6 eV, respectively.

**Fig. 4:**
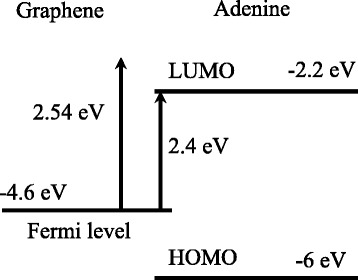
The energy level scheme of the graphene-adenine system

If we suppose that the graphene Fermi level is situated near −4.6 eV [25], we can finally construct an approximate energy level scheme of the graphene-adenine system (Fig. 4). From the latter, the 2.41-eV photon energy is in a better resonance with the Fermi level-LUMO transition compared to the 2.54-eV photons.

Voltage-dependent shift of the graphene’s Fermi level could be another way to control the resonance conditions for electron transfer to adenine on graphene. Our first attempts in this direction were not very successful because graphene suffers from hysteresis of electrical characteristics in atmospheric conditions [25]. It complicates the electrical control of the graphene’s Fermi level.

## Conclusions

Enhanced Raman scattering from thin adenine layers deposited on graphene was detected. It was accompanied by quenching of the background fluorescence of adenine by the underlying graphene layer. The enhancement of the Raman signal depends on the photon energy of the exciting light in a manner which is in agreement with the assumption that a resonant electron transfer from the Fermi level of graphene to the LUMO level of adenine molecule takes place.

## References

[1] Boiko V, Fesenko O, Gorchev V, Karakhim S, Dolgov L, Kiisk V, Sildos I, Gorelik V, Dovbeshko G, Fesenko O, Yatsenko L, Brodin M (2013). Luminescent imaging of biological molecules and cells on the photonic crystal surface. Nanomaterials imaging techniques, surface studies and applications.

[2] Stockman MI, Kneipp K, Moskovits M, Kneip H (2006). Electromagnetic theory of SERS. Surface-enhanced Raman scattering physics and applications.

[3] Schlücker S (2014). Surface-enhanced Raman spectroscopy: concepts and chemical applications. Angew Chem Int Edit.

[4] Otto C, Mul FFM, Huizinga A, Greve J (1988). Surface enhanced Raman scattering of derivatives of adenine: the importance of the external amino group in adenine for surface binding. J Phys Chem.

[5] Otto C, Hoeben F, Greve J (1991). Complexes of silver with adenine and dAMP. J Raman Spectros.

[6] Jha S, Ahmed Z, Agio M, Ekinci Y, Loffler J (2012). Deep-UV surface-enhanced resonance Raman scattering of adenine on aluminum nanoparticle arrays. J Am Chem Soc.

[7] Mevold A (2015). Fabrication of gold nanoparticles/graphene-PDDA nanohybrids for biodetection by SERS nanotechnology. Nanoscale Res Lett.

[8] Xi L (2010). Can graphene be used as a substrate for Raman enhancement?. Nano Lett.

[9] Xu W, Mao N, Zhang J (2013). Graphene: a platform for surface-enhanced Raman spectroscopy. Small.

[10] Huang S (2015). Molecular selectivity of graphene-enhanced Raman scattering. Nano Lett.

[11] Jablan M, Soljacic M, Buljan H (2013). Plasmons in graphene: fundamental properties and potential applications. P IEEE.

[12] Zhang D (2003). Raman detection of proteomic analytes. Anal Chem.

[13] Skuja L (1998). Optically active oxygen-deficiency-related centers in amorphous silicon dioxide. J Non-Cryst Solids.

[14] Galeener F (1979). Band limits and the vibrational spectra of tetrahedral glasses. Phys Rev B.

[15] Galeener F, Geissberger A (1983). Vibrational dynamics in ^30^Si-substituted vitreous SiO_2_. Phys Rev B.

[16] Galeener F, Lucovsky G (1976). Longitudinal optical vibrations in glasses: GeO_2_ and SiO_2_. Phys Rev Lett.

[17] Russell J (1965). Raman scattering in silicon. Appl Phys Lett.

[18] Parker J, Feldman D, Ashkin M (1967). Raman scattering by silicon and germanium. Phys Rev.

[19] Ferrari A, Basko D (2013). Raman spectroscopy as a versatile tool for studying the properties of graphene. Nat Nanotech.

[20] Mathlouthi M, Seuvre A-M (1984). F.T.-I.R. and laser-Raman spectra of adenine and adenosine. Carbohyd Res.

[21] Silaghi S, Friedrich M, Cobet C, Esser N, Braun W, Zahn D (2005). Dielectric functions of DNA base films from near-infrared to ultra-violet. Phys Stat Sol B.

[22] Mishra S, Shukla M, Mishra P (2000). Electronic spectra of adenine and 2-aminopurine: an ab initio study of energy level diagrams of different tautomers in gas phase and aqueous solution. Spectrochim Acta A.

[23] Kilina S, Tretiak S, Yarotski D, Zhu J, Modine N, Taylor A, Balatsky A (2007). Electronic properties of DNA base molecules adsorbed on a metallic surface. J Phys Chem C.

[24] Faber C, Attaccalite C, Olevano V, Runge E, Blase X (2011). First-principles GW calculations for DNA and RNA nucleobases. Phys Rev B.

[25] Xu H, Xie L, Zhang H, Zhang J (2011). Effect of graphene Fermi level on the Raman scattering intensity of molecules on graphene. ACS Nano.

